# A Sparse EEG-Informed fMRI Model for Hybrid EEG-fMRI Neurofeedback Prediction

**DOI:** 10.3389/fnins.2019.01451

**Published:** 2020-01-31

**Authors:** Claire Cury, Pierre Maurel, Rémi Gribonval, Christian Barillot

**Affiliations:** ^1^University of Rennes, CNRS, Inria, Inserm, IRISA UMR 6074, Empenn Team ERL U 1228, Rennes, France; ^2^University of Rennes, CNRS, Inria, IRISA UMR 6074, PANAMA Team, Rennes, France

**Keywords:** optimization, EEG, sparsity, machine learning, neurofeedback, EEG-fMRI

## Abstract

Measures of brain activity through functional magnetic resonance imaging (fMRI) or electroencephalography (EEG), two complementary modalities, are ground solutions in the context of neurofeedback (NF) mechanisms for brain rehabilitation protocols. While NF-EEG (in which real-time neurofeedback scores are computed from EEG signals) has been explored for a very long time, NF-fMRI (in which real-time neurofeedback scores are computed from fMRI signals) appeared more recently and provides more robust results and more specific brain training. Using fMRI and EEG simultaneously for bi-modal neurofeedback sessions (NF-EEG-fMRI, in which real-time neurofeedback scores are computed from fMRI and EEG) is very promising for the design of brain rehabilitation protocols. However, fMRI is cumbersome and more exhausting for patients. The original contribution of this paper concerns the prediction of bi-modal NF scores from EEG recordings only, using a training phase where EEG signals as well as the NF-EEG and NF-fMRI scores are available. We propose a sparse regression model able to exploit EEG only to predict NF-fMRI or NF-EEG-fMRI in motor imagery tasks. We compared different NF-predictors stemming from the proposed model. We showed that predicting NF-fMRI scores from EEG signals adds information to NF-EEG scores and significantly improves the correlation with bi-modal NF sessions compared to classical NF-EEG scores.

## 1. Introduction

Neurofeedback approaches (NF) provide real-time feedback to a subject about his or her brain activity and help him or her perform a given task (Hammond, 2011; Sulzer et al., 2013). The estimation of neurofeedback information is done through online brain functional feature extraction to provide this real-time feedback to the subject. NF appears to be an interesting approach for clinical purposes, for example, in the context of rehabilitation and psychiatric disorders (Birbaumer et al., 2009; Sulzer et al., 2013; Wang et al., 2017). Functional magnetic resonance imaging (fMRI) and electroencephalography (EEG) are the most used non-invasive functional brain imaging modalities in neurofeedback. EEG measures the electrical activity of the brain through electrodes located on the scalp. EEG has an excellent temporal resolution (milliseconds) but a limited spatial resolution (centimeters), implying a lack of specificity. Furthermore, source localization in EEG is a well-known ill-posed inverse problem (Grech et al., 2008).

On the other hand, blood oxygenation level-dependent (BOLD) fMRI measures a delayed hemodynamic response to neural activity with a good spatial resolution and a temporal resolution of 1 or 2 s depending on the sequence used. Therefore, fMRI is more specific than EEG, making fMRI a suitable modality for neurofeedback (NF-fMRI) (Thibault et al., 2018). However, the use of the MRI scanner is costly, exhausting for patients (since staying perfectly still when suffering is challenging), and time-consuming, and hence NF-fMRI sessions cannot be repeated an excessive number of times for the same subject or patient.

During the past few years, simultaneous EEG-fMRI recording has been used to understand the links between EEG and fMRI in different states of brain activity and has received recognition as a promising multi-modal measurement of brain activity (Abreu et al., 2018; Perronnet et al., 2018). However, this bi-modal acquisition is cumbersome for subjects or patients due to the use of the fMRI scanner. The methodology to extract information from fMRI with EEG has been also intensively investigated (some methods involved in the process are reviewed in Abreu et al., 2018). Indeed, both modalities are sensitive to different aspects of brain activity with different speeds. EEG provides a direct measure of the changes in electrical potential occurring in the brain in real time, while fMRI indirectly estimates brain activity by measuring changes in the BOLD signal, reflecting neuro-vascular activity, which occurs, in general, a few seconds after a neural event (Friston et al., 1994; Logothetis et al., 2001). Several studies have investigated correlations between EEG signal and BOLD activity in specific and simple tasks (de Munck et al., 2007; Goncalves et al., 2008; Scheeringa et al., 2011; Engell et al., 2012; Magri et al., 2012) and have found different relationships of certain frequency bands of the EEG signal. All those studies reveal the existence of a link between EEG and fMRI, but this relationship varies considerably with the task, location in the brain, and frequency bands considered.

In the literature, the term EEG-informed fMRI refers to methods extracting features from EEG signals in order to derive a predictor of the associated BOLD signal in the region of interest under study. A recent review (Abreu et al., 2018) gives a good overview of the principal EEG-informed fMRI methods and their limitations. Different strategies have been investigated, depending on the type of activity under study (epilepsy, resting state, open/closed eyes, relaxation): either selecting one channel of interest or using multiple channels before extracting features of interest. For example, in Formaggio et al. (2011) and Leite et al. (2013), the authors used a temporal independent component analysis to select the channel that best reflected epileptic seizures. In Schwab et al. (2015), the authors used a spatial, spectral, and temporal decomposition of the EEG signals to map EEG onto BOLD signal changes in the thalamus. Using a more symmetrical approach, Noorzadeh et al. (2017) proposed a method for the estimation of brain source activation, improving the spatio-temporal resolution compared to EEG or BOLD fMRI only. However, in the context of neurofeedback, using simultaneous recording of EEG-fMRI to estimate neurofeedback scores computed in real time from features of both modalities (NF-EEG-fMRI) is a recent application that was first introduced and its feasibility demonstrated by Zotev et al. (2014), Mano et al. (2017), and Perronnet et al. (2017). The recent methodology synchronizing both signals for real-time neurofeedback (Mano et al., 2017) allows the construction of a new kind of data named NF-EEG-fMRI data, such as the dataset presented by Perronnet et al. (2017), which we used for the present study. Furthermore, it has been shown in Perronnet et al. (2017) that the quality of a neurofeedback session is improved when using both modalities simultaneously in NF-EEG-fMRI sessions. Thus, being able to reproduce an NF-EEG-fMRI session in real time when using EEG only would reduce the use of fMRI in neurofeedback while increasing the quality of NF-EEG sessions. To export fMRI information outside the scanner, most of the methods intend to predict the fMRI BOLD signal activity in a specific region of interest by learning from an EEG signal recorded simultaneously inside the fMRI scanner. Indeed, the method proposed in Meir-Hasson et al. (2014) uses a ridge regression model with a ℓ_2_ regularization, based on a time/frequency/delay representation of the EEG signal from a single channel. Results show a good estimation of the BOLD signal in the region of interest, but the use of the fMRI neurofeedback in this study is only to serve the paradigm. The method aims at better targeting the amygdala in NF-EEG sessions.

**Figure 1:**
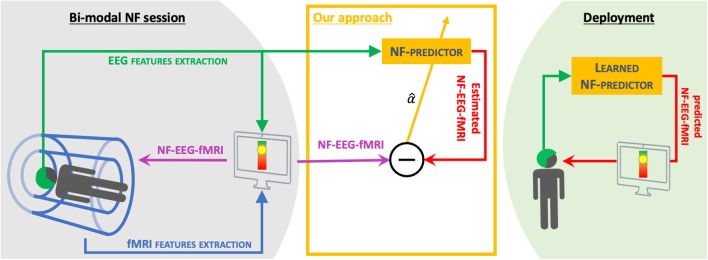
Objective: We propose a method of learning a NF-predictor from bi-modal neurofeedback sessions (NF-EEG-fMRI) (see Perronnet et al., 2017 or section 3 for more details). The final goal of this method is to propose NF sessions using EEG only that have the quality of bi-modal NF sessions, therefore reducing the use of fMRI.

Our challenge here is to learn EEG activation patterns (see section 2.2) from hybrid (or bi-modal) NF-EEG-fMRI sessions (Perronnet et al., 2017) and improve the correlation with NF-EEG-fMRI of NF scores using the EEG signal only. The motivations of this are multiple. Since we are considering a new kind of data, we want to provide a simple method of characterizing NF-EEG-fMRI in EEG, leading to an understandable model to confirm existing relations between EEG and fMRI in neurofeedback scores or to discover new relationships. Neurofeedback features in fMRI come from BOLD activation in one or more regions of interest. We propose an original alternative to source reconstruction in the context of neurofeedback by taking advantage of a recent and unique dataset of NF-EEG-fMRI that we have access to. Indeed, we intend to predict NF scores directly without dealing with source reconstruction or spatial filtering to estimate the BOLD-fMRI signal first in a specific region of interest, as proposed by a previous study (Noorzadeh et al., 2017). To our knowledge, this prediction of hybrid neurofeedback scores (without source reconstruction) is new and has not yet been explored in the literature. Also, we want the activation pattern to be applicable in real time when using new EEG data. The main objective of this paper (Figure 1) is to design a method able to exploit EEG only and predict an NF score of a quality comparable to the NF score that could be achieved with a simultaneous NF-EEG-fMRI session. The approach is based on a machine learning mechanism. During a training phase, both EEG and fMRI are simultaneously acquired and used to compute and synchronize, in real time, NF-EEG and NF-fMRI scores, both being combined into a hybrid NF-EEG-fMRI score (Mano et al., 2017). EEG signals and NF scores are used to learn activation patterns. During the testing phase, the learned NF-predictor (also called the activation pattern) is applied to unseen EEG data, providing simulated NF-EEG-fMRI scores in real time. Sparse regularization is exploited to build a model called the NF-predictor. The model used for the NF-predictor uses an adapted prior for brain activation patterns, using a mixed norm giving a structured sparsity, to spatially select electrodes and then select the most relevant frequency bands.

In section 2, we present the proposed model and the methods used to solve it. We then detail experiments with our learning model in neurofeedback sessions with a motor imagery task, from which unique data are presented in section 3. Section 4 presents results for a dataset of 17 healthy subjects with three NF sessions of motor imagery each; one is used to learn the model, and the other two are used to test the model. Section 5 provides a discussion of the proposed framework.

## 2. Problem and Method

Considering that, during a learning phase, we have access to reference scores *y*(*t*) and a temporal representation (potentially non-linear) of EEG signals ***X*** (called a design matrix, presented in section 2.1), the approach consists of choosing a vector of parameters **α** such that *y*(*t*) ≈ *q*(***X***(*t*), ***α***) for all *t*, where *q* is some parametric function. ***α*** is a matrix matching the size of ***X***(*t*). Here we consider

(1) $$\begin{array}{l} qX\left(t\right),\alpha ):=\langle X\left(t\right),\alpha \rangle =\overset{E}{\underset{i=1}{\sum }}\overset{F}{\underset{j=1}{\sum }}X_{i,j}\left(t\right)\alpha_{i,j}. \end{array}$$

**Figure 2:**
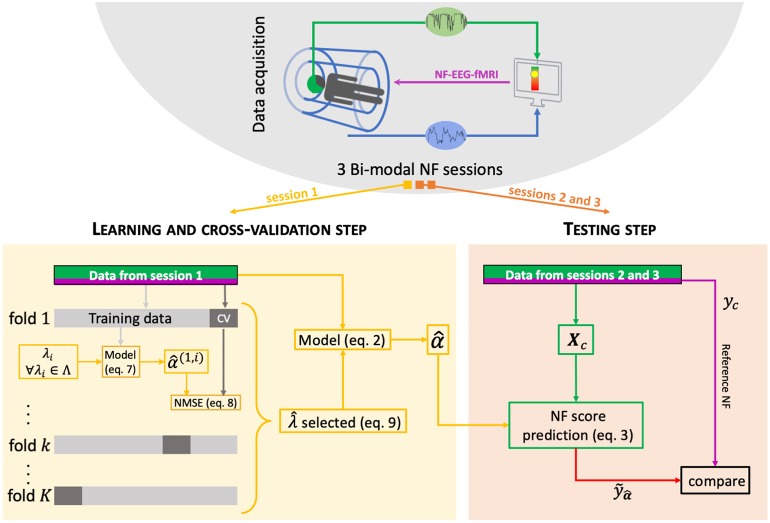
Machine learning scheme. For each subject, a bimodal neurofeedback session (NF-EEG-fMRI session 1 here) is used for the learning step, and then the learned activation pattern $\hat{\alpha }$ is applied to the other sessions (2 and 3) for the testing step. The learning data are split *K* times into a training set (90% of the learning set) and a cross-validation (CV) set (10% of the learning set). The optimal $\hat{\lambda }$ parameter is the one minimizing the variance and the bias in the learning step.

To determine this parameter ***α***, a regularization is used to select an optimal parameter $\hat{\alpha }$ that fits the training data (see Figure 2 and section 2.3) while avoiding over-fitting, as detailed in section 2.2.

Only a few brain regions are expected to be activated by a given cognitive task, so the electrode configuration is said to be spatially sparse. However, the frequency bands of each electrode are not necessarily sparse and might even be smooth, depending on the frequency band sampling.

From here, we will use the following notations:

- *y*_e_(*t*) ∈ ℝ, ∀*t* ∈ {1, …, *T*} are the *T* neurofeedback scores estimated from EEG signals (noted *S*_EEG_ ∈ ℝ^*E*×*T*_EEG_^) measured from *E* electrodes during *T*_EEG_ samples of time.
- *y*_f_(*t*) ∈ ℝ, ∀*t* ∈ {1, …, *T*} are the *T* neurofeedback scores extracted from the Blood Oxygen Level Dependent imaging (BOLD) signal of functional-MRI acquisitions *S*_fMRI_ ∈ ℝ^*V*×*T*_fMRI_^, with *V* the number of voxels and *T*_fMRI_ the number of acquired volumes.
- *y*_c_(*t*) = *y*_e_(*t*)+*y*_f_(*t*) ∈ ℝ, ∀*t* ∈ {1,…,*T*}, a combination of both NF scores (more details are provided in section 3).
- *y*(*t*) ∈ ℝ, ∀*t* ∈ {1,…,*T*} is a set of neurofeedback scores that can be *y*_e_, *y*_f_, or *y*_c_.

First, to build our predictor, relevant information needs to be extracted from EEG data and organized to form what we call a design matrix.

### 2.1. Structured Design Matrices From the EEG Signal

The design matrix ${\text{X}}_{0}\in {\mathbb{R}}^{T\times E\times B}$, where *E* is the number of electrodes and *B* the number of frequency bands, contains relevant information extracted from the EEG signal. Each temporal matrix of ***X***_0_, ${\text{X}}_{0}\left(t\right)\in {\mathbb{R}}^{E\times B}\forall t\in \left\{1;\ldots ;T\right\}$ is a frequency decomposition corresponding to the past 2 s of *S*_EEG_. We used a Hamming time window of 2 s to estimate the average power of each frequency band *b* ∈ {1;…;*B*} (defined below) on each channel ∈ {1;…;*E*}. Each time window of the EEG signal overlapped by 1.75 s (0.25-s shift) to match with the 4 Hz sample of the ***y*** values. The *B* frequency bands have an overlap of 1 Hz with the next band and are defined between a minimum frequency *b*_*min*_ Hz and a maximum frequency *b*_*max*_ Hz (see section 3). We chose to use several relatively narrow frequency bands to let the model select the relevant bands for each electrode. Furthermore, it has been suggested (de Munck et al., 2009; Rosa et al., 2010) that different frequency bands be used when working with coupled EEG-fMRI data.

The model also has to be able to predict *y*_f_ scores derived from the BOLD signal (see section 3). There is no linear relationship between the BOLD signal and the average power on frequency bands from the EEG signal. Therefore, to better match *y*_f_ scores, we decided to apply a non-linear function to ***X***_0_ used in fMRI to model BOLD signals (Lindquist et al., 2009; Pedregosa et al., 2013), the canonical Hemodynamic Response function (HRF). We convolved ***X***_0_ on its temporal dimension with the HRF, formed by two gamma functions, for a given delay of the first gamma function to compensate the response time of the BOLD signal, as suggested in Moosmann et al. (2008) and Meir-Hasson et al. (2014). The HRF will temporally smooth and give a BOLD-like shape to the design matrix and increase a potential linear relationship between *y*_f_ and the design matrix. Since HRF is known to vary considerably across brain regions and subjects (Handwerker et al., 2004), it is recommended to consider different delays but also to chose a range of values corresponding to the task asked. For the type of task addressed in the experimental part, the observed delay is around 4 s, so we convolved X0 with three different HRFs, leading to three new design matrices, ***X***_3_, ***X***_4_, *and* ***X***_5_, with respectively peak locations of 3, 4, and 5 s. We started from the canonical HRF and changed the peak parameter (respectively using 3, 4, and 5) to induce the three different HRFs used to induce a time delay in the initial design matrix ***X***_0_. By doing so, we kept the total of design matrices to a reasonable size.

These design matrices are concatenated in their 2nd dimension to form the ${\text{X}}_{c}\in {\mathbb{R}}^{T\times M\times B}$ matrix, with *M* = 4 * *E*. Therefore, for each time *t*, ***X***_*c*_(*t*) = [***X***_0_(*t*); ***X***_3_(*t*); ***X***_4_(*t*); ***X***_5_(*t*)]. We also denote ***X***_*d*_(*t*) = [***X***_3_(*t*); ***X***_4_(*t*); ***X***_5_(*t*)] the design matrix of the different delays.

### 2.2. Optimization

The EEG data are now represented by a structured design matrix ***X***_*c*_, and we can search for a weight matrix $\hat{\alpha }\in {\mathbb{R}}^{M\times B}$, such that $\underset{m,h}{\sum }\hat{\alpha }\left(m,h\right){\text{X}}_{c}\left(t,m,h\right)$ estimates the NF score *y*(*t*), ∀*t* ∈ {1;…;*T*} as well as possible. Note: the methodology is presented for the design matrix ***X***_*c*_ but can be used for ***X***_0_ or ***X***_*d*_.

To identify $\hat{\alpha }$, called the activation pattern, we propose the following strategy, which consists of learning, for a given subject and NF session, the optimal $\hat{\alpha }$ by solving the following problem:

(2) $$\hat{\alpha }=\;\underset{\alpha }{\text{argmin}}\;\overset{T}{\underset{t=1}{\sum^{\;}}}\frac{1}{2}{\left(y(t)-q(X_{c}(t),\alpha \right))}^{2}+\;\phi_{\lambda }(\alpha )$$

with ϕ_λ_ a regularization term, and λ a weighting parameter for the regularization term. This $\hat{\alpha }$ is then applied to a design matrix ${\text{X}}_{c}^{test}$ from a new session to predict its NF scores.

(3) $${\tilde{y}}_{\hat{\alpha }}(t)=q({X_{c}}^{test}(t),\hat{\alpha })\forall t\in \{1;\ldots ;T\}$$

Equation 2 is of the form argmin(*g*_1_(***α***)+*g*_2_(***α***)), and its resolution can be performed using the Fast Iterative Shrinkage Thresholding Algorithm (FISTA) (Beck and Teboulle, 2009), which is a two-step approach of the Forward-Backward algorithm (Combettes and Wajs, 2005), making it faster. FISTA requires the same conditions as the Forward-Backward algorithm, meaning a convex differentiable with Lipschitz gradient term *g*_1_ and a convex term *g*_2_ that is not necessarily differentiable but smooth enough to make its proximal map computable.

Here, $g_{1}\left(\alpha \right)=\overset{T}{\underset{t}{\sum }}\frac{1}{2}{\left(\text{y}\left(t\right)-q\left({\text{X}}_{c}\left(t\right),\alpha \right)\right)}^{2}$ is a sum of convex and differentiable functions with

(4) $$\begin{array}{l} ▽g_{1}\left(\alpha \right)=\underset{t}{\sum }-X_{c}\left(t\right)\left(y\left(t\right)-q\left(X_{c}\left(t\right),\alpha \right)\right) \end{array}$$

since $\forall i\in \left\{1;\ldots ;M\right\},j\in \left\{1;\ldots ;B\right\},{\left[\frac{\partial q\left({\text{X}}_{c}\left(t\right),\alpha \right)}{\partial \alpha \left(i,j\right)}\right]}_{i,j}={\text{X}}_{c}\left(t,i,j\right)$. By representing ***X***_*c*_(*t*) and ***α*** as vectors of size *M* * *B*, we can easily note that $\frac{\partial g_{1}}{\partial \alpha }$ is a sum of Lipschitz functions. Therefore, the Lipschitz constant of $\frac{\partial g_{1}}{\partial \alpha }$ is $L=\|X_{V}^{⊺}X_{V}\|$, with $X_{V}\in {\mathbb{R}}^{T*M*B}$ the vectorized version of ***X***_*c*_.

The NF-predictor uses the structured design matrix to achieve better control over the interpretation of results and to better optimize the weights $\hat{\alpha }$. Therefore, we have to adopt an optimization strategy that is coherent with this structure. The activation pattern of the NF-predictor:

1. has to be spatially sparse to regulate the model, as EEG signals are noisy, and to select the most relevant electrodes on each frequency bands,
2. has to be smooth across different overlapped frequency bands,
3. has to allow non-relevant frequency bands to be null.

The term *g*_2_ is the prior term. Here, for *g*_2_(***α***) = ϕ_λ_(***α***), we chose to use a ℓ_21_ mixed norm (Ou et al., 2009) followed by a ℓ_1_-norm (noted ℓ_21+1_-norm in Gramfort et al., 2011) to fit all the structure conditions mentioned above.

(5) $$\begin{array}{l} \phi_{\lambda }\left(\alpha \right)=\lambda \|\alpha {\|}_{21}+\rho {\left\|\alpha \right\|}_{1} \end{array}$$

with ρ ∈ ℝ^+^ and λ ∈ ℝ^+^. We chose not to estimate the parameter ρ to keep computation time reasonable. Indeed, ρ weights the induced spatial sparsity over EEG channels, and we chose to fix this parameter for all subjects, as we hypothesize that there is no reason for the number of electrodes involved in the activation pattern to significantly change between subjects. However, the estimation of the λ parameter is needed (since we do not have a hypothesis on its behavior) and is presented in the next section. The ℓ_21_ mixed norm that writes $\|\alpha {\|}_{21}=\underset{m}{\sum }\sqrt{\underset{b}{\sum }\alpha_{m,b}^{2}}$ satisfies conditions (1) and (2). The ℓ_1_ norm defined as $\left\|\alpha {\|}_{1}\;=\;\underset{m,b}{\sum }|\alpha_{m,b}\right|$ satisfies condition (3) since ℓ_*p*_ norms with *p* ≤ 1 are known to promote sparsity. The last key point of FISTA algorithm is the proximal map associated to the ℓ_21+1_ norm ${\text{Prox}}_{\ell_{21+1}}:{\mathbb{R}}^{M\times B}\to {\mathbb{R}}^{M\times B},\beta \mapsto \text{argmi}{\text{n}}_{\alpha }\left(\phi_{\lambda }\left(\alpha \right)+1/2{\left\|\beta -\alpha \right\|}^{2}\right)$, defined as

(6) $$\begin{array}{l} {\left({\text{Prox}}_{\ell_{21+1}}\left(Y\right)\right)}_{m,b}\\ =\frac{Y_{m,b}}{\left|Y_{m,b}\right|}{\left(|Y_{m,b}|-\rho \right)}^{+}{\left(1-\frac{\lambda }{\sqrt{\sum_{b}{\left(|Y_{m,b}|-\rho \right)}^{+2}}}\right)}^{+} \end{array}$$

with operator (.)^+^ = *max*(.,0). One can note that by canceling either the λ parameter or the ρ parameter, we retrieve the proximal map associated with the ℓ_21_ (when ρ = 0) and the ℓ_1_ (when λ = 0) norms, demonstrations of which can be found in the appendix of Gramfort et al. (2012). For the stopping criterion of FISTA, a large enough number of iterations has been used to allow the model to converge before reaching the last iteration. All elements and conditions are gathered to run the FISTA algorithm.

### 2.3. λ Parameter Selection

The parameter λ is important in the optimization problem, and we decided to estimate it automatically. The following process chooses the best λ among a list of Λ = {λ_1_; …;λ_*l*_} sorted in increasing order. First of all, the data must be split into two sets. In our case, subjects have three NF-EEG-fMRI sessions: one session is used as the learning set, and the other two NF-EEG-fMRI sessions are used as the testing sets (see Figure 2). For each value λ_*i*_ of Λ, the learning set, formed by *T* neurofeedback scores with their associated design matrices, is divided *K* = 50 times into a training set of indices *R*_*k*_, representing 90% of the *T* data, and a cross-validation set *CV*_*k*_ composed of the remaining 10% of the learning set. A model ${\hat{\alpha }}^{\left(k,i\right)}$ is estimated on the training dataset *k* composed by *R*_*k*_ neurofeedback scores *y*(*j*) and the associated design matrices ***X***_*c*_(*j*) with λ_*i*_, i.e.:

(7) $$\begin{array}{lll} {\hat{\alpha }}^{\left(k,i\right)} & = & \arg\min\underset{j}{\sum }\|y\left(j\right)-q\left(X_{c}\left(j\right),{\hat{\alpha }}^{\left(k,i\right)}\right){\|}^{2}\\ \; & + & \lambda_{i}\|\alpha^{\left(k,i\right)}{\|}_{21}+\rho \|\alpha^{\left(k,i\right)}{\|}_{1} \end{array}$$

with *j* ∈ *R*_*k*_ and ${\text{X}}_{c}\left(j\right)\in {\mathbb{R}}^{M\times B}$. For the current λ_*i*_ evaluation, we stop the process when $\underset{k}{\sum }|{\hat{\alpha }}^{\left(k,i\right)}{|}_{0}/K<2$. There is no need to investigate the next λ_*i*_; the current one is sparse enough, and the next one might lead to null models.

We then apply the model ${\hat{\alpha }}^{\left(k,i\right)}$ to the corresponding cross-validation set of *CV*_*k*_ NF scores to obtain estimated values of *y*(*s*), $\tilde{y}\left(s\right)=q\left(X_{c}\left(s\right),{\hat{\alpha }}^{\left(k,i\right)}\right)$ with *s* ∈ *CV*_*k*_. For each one of the 50 partitioned into training and cross-validation sets, we computed the normalized mean squared error NMSE for a given set of data {*y*, ***X***_*c*_} for the training sets and the cross-validation sets.

(8) $$\begin{array}{ll} \text{NMSE}\left(\left\{y,X_{c}\right\},{\hat{\alpha }}^{\left(k,i\right)}\right) & =\frac{\sum_{s}{\left(y\left(s\right)-q\left(X_{c}\left(s\right),{\hat{\alpha }}^{\left(k,i\right)}\right)\right)}^{2}}{\sum_{s}{\left(y\left(s\right)-\bar{y}\right)}^{2}} \end{array}$$

with $\bar{\text{y}}=1/n\overset{n}{\underset{s}{\sum }}y\left(s\right)$. The optimal $\hat{\lambda }$ parameter is defined as the one minimizing the error during training and cross-validation. Considering that only the errors from the training set $\text{NMSE}\left(\left\{y\left(R_{k}\right),{\text{X}}_{c}\left(R_{k}\right)\right\},{\hat{\alpha }}^{\left(k,i\right)}\right)$ would introduce bias, and considering only the error of the cross-validation set $\text{NMSE}\left(\left\{y\left(CV_{k}\right),{\text{X}}_{c}\left(CV_{k}\right)\right\},{\hat{\alpha }}^{\left(k,i\right)}\right)$ would introduce variance. Then, the optimal $\hat{\lambda }$ is the λ_*i*_ that minimizes

(9) $$\begin{array}{l} \;\sum_{k=1}^{k}[\text{NMSE}(\{y(R_{k}),X_{c}(R_{k})\},{\hat{\alpha }}^{\left(k,i\right)})\\ +\text{NMSE}(\{y(CV_{k}),X_{c}(CV_{k})\},{\hat{\alpha }}^{\left(k,i\right)})] \end{array}$$

The $\hat{\lambda }$ parameter is the optimal parameter used for the model estimation. If there are several candidates, to favor sparsity, the larger of these candidates is chosen.

## 3. Data Acquisition and Pre-processing

**Figure 3:**
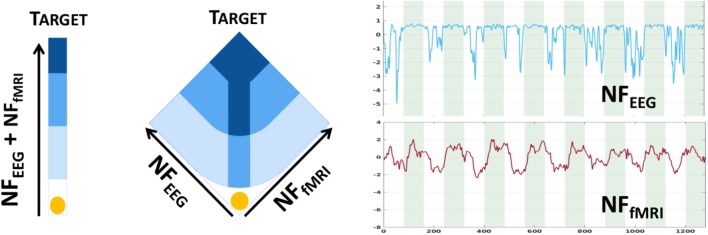
Bi-modal neurofeedback metaphors (1D on the left, 2D on the middle) displayed during sessions (Perronnet et al., 2018). 1D: the ball's position represents the sum of *y*_e_ and *y*_f_. 2D: the left axis represents the *y*_e_, and the right axis represents the *y*_f_ scores. The two plots on the right show NF scores from EEG and from fMRI; green areas are task and white areas are rest. The goal is to bring the ball into the dark blue area.

We used an existing dataset presented in Perronnet et al. (2018) composed of 17 healthy subjects who were scanned using the hybrid Neurofeedback platform from Neurinfo (Rennes, France) coupling EEG and fMRI signals (Mano et al., 2017). Data are now available online in BIDs format on OpenNeuro: https://openneuro.org/datasets/ds002338 (Lioi et al., 2019). A 64-channel MR-compatible EEG solution from Brain Products (Brain Products GmbH, Gilching, Germany) was used, the signal was sampled at 5 kHz, and FCz was the reference electrode and AFz the ground electrode. For the fMRI scanner, we used a 3T Verio Siemens scanner with a 12-channel head coil (repetition time (TR)/echo time (TE) = 2,000/23 ms, FOV = 210 × 210 mm^2^, voxel size = 2 × 2 × 4 mm^3^, matrix size =105 × 105 with 16 slices, flip angle = 90°). All subjects were healthy volunteers, were right-handed, and had never taken part in any neurofeedback experiments before. They all gave written informed consent in accordance with the Declaration of Helsinki, as specified in the study presenting the data (Perronnet et al., 2017). They all underwent three NF motor imagery sessions of 320 s each after a session dedicated to the calibration. One session consists of eight blocks alternating between 20 s of rest, eyes open, and 20 s of motor imagery of the right hand. The neurofeedback display was uni-dimensional (1D) for nine subjects (Figure 3 left) and bi-dimensional (2D) for eight subjects (Figure 3 middle). For both, the goal was to bring the ball into the dark blue area (Perronnet et al., 2018).

NF scores *y*_e_ and *y*_f_, being from different modalities, were standardized before summing to form *y*_c_. In this study, NF scores refer to standardized scores except when they are predicted. For this study, *y*_e_ have been computed from the Laplacian operator (commonly used in neurofeedback) centered around the region of interest, channel C3 here. For each time interval *I*_*t*_, the spatial filtering is noted as Lap(C3, *I*_*t*_). The temporal segments *I*_*t*_ are spaced at 250 ms apart and have a length of 2 s (therefore, there is an overlap of 1.75 s), as in the design matrix construction. The power of the frequency band [8–30 Hz] is then extracted via the Power Spectral Density function *PSD*:

$$y_{e}(t)=-PSD_{\left[8-30\right]}(\text{Lap(C3,}I_{t}))$$

One may note the presence of the minus operator, which is used here for the sake of coherence with *y*_f_ (Figure 3 right).

The neurofeedback scores *y*_f_ have been computed from the maximal intensity of BOLD signal covering the right-hand motor area and the supplementary motor area. One score is computed per volume acquired (i.e., 1 per second). Scores *y*_f_ are then re-sampled and smoothed (using a Savitzky-Golay filter, known to avoid signal distortion) to fit the 4 Hz *y*_e_ scores (*T* = 1, 280).

An active set has been selected on the design matrices to avoid potentially correlated noise due to head movement during resting blocks obstructing the signal from channels of interest. Indeed in coupled EEG-fMRI acquisitions, subjects are lying in the MRI scanner, so the outer electrodes may be in contact with the bed or holds. We excluded the outer electrodes and kept 28 electrodes: the three central lines have seven electrodes (FCz is the reference), three frontal electrodes, and three posterior electrodes.

Potential outliers in the design matrices (i.e., observations > mean ± 3std) were thresholded in the NF-EEG-fMRI session used as the learning set, and bad observations from annotations on the EEG signal were removed as their corresponding NF scores. For the frequency dimension of the design matrix ***X***_0_ construction (cf. section 2) and therefore for the other design matrices, we chose *b*_*min*_ = 8, *b*_*max*_ = 30 to cover the alpha and beta frequency bands involved in motor tasks. We considered *B* = 10 frequency bands, leading to bands of 3 Hz wide with an overlap of 1Hz.

As mentioned in the previous section, for the regularization of the NF-predictor, we used 15 values of λ_*i*_ from 100 to 3,000 and fixed the parameter ρ = 1, 500.

## 4. Experiments and Results

As stated in the previous section, there were three neurofeedback sessions per subject. For each subject, we will consider one session as a learning set, and the two others as testing sets. Thus, there are three different learning sets and six different testing sets per subject.

### 4.1. Experiments and Validation

We tested different NF-predictors for the prediction of different NF scores, ∀*t* ∈ {1;…;*T*}:

1. ${\tilde{y}}_{{\hat{\alpha }}_{c}}(t)=q(X_{c}^{test}(t),\;\alpha_{c})$ with ${\hat{\alpha }}_{c}$ (Equation 2), learned from ***X***_*c*_ and *y*_c_
2. ${\tilde{y}}_{{\hat{\alpha }}_{f}}(t)=q(X_{d}^{test}(t),\;\alpha_{f})$ with ***X***_*d*_ = [***X***_3_; ***X***_4_; ***X***_5_] and ${\hat{\alpha }}_{f}$ (Equation 2), learned from ***X***_*d*_ and *y*_f_
3. $y_{\text{e}}(t)+{\tilde{y}}_{{\hat{\alpha }}_{f}}$ (*t*) using NF-predictor 2.

The advantage of the last NF-predictor is the possibility of using the 2D score visualization (Figure 3) to display NF scores. Also in this case, only NF-fMRI scores have to be learned. We run the following experiments to test our different NF-predictors:

- Model validation: we used the learning set to assess whether the different NF-predictors could accurately model the NF scores. For each subject and each NF-predictor, we estimated correlations with the reference NF score to quantify the quality of prediction. As a reference, we compared to the correlation of *y*_e_ to *y*_c_, which is part of the *y*_c_ score, to assess whether, in the validation process, the model can better predict *y*_c_ scores than *y*_e_.
- Model prediction: we applied the learned activation patterns to a new NF-EEG-fMRI recorded session (testing set, Figure 2). For each subject and each pair of sessions (six learning/testing pairs per subject), we compared correlations between NF-predictors ${\tilde{y}}_{{\hat{\alpha }}_{c}}$ and $y_{\text{e}}+{\tilde{y}}_{{\hat{\alpha }}_{f}}$ to reference score *y*_c_ and between the prediction of ${\tilde{y}}_{{\hat{\alpha }}_{f}}$ to *y*_f_.
- To observe the captured structure in the activation patterns ${\hat{\alpha }}_{c}$ and ${\hat{\alpha }}_{f}$, respectively learned to predict *y*_c_ and *y*_f_, we re-shaped the average activation patterns of a subject over sessions into matrices corresponding to the design matrices defining ***X***_*c*_, respectively ***X***_*d*_ (see section 2.1) and displayed the results of the first dimension (electrodes) and of the second dimension (frequency bands).

### 4.2. Results

#### 4.2.1. Model and Prediction

**Figure 4:**
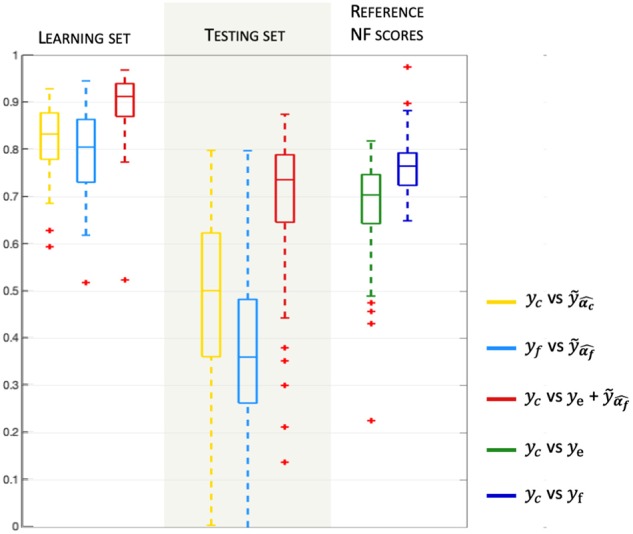
Model validation and prediction. Boxplots (median and quartiles) of Pearson's correlation coefficients, over all subjects and sessions, between NF-predictors and *y*_c_ or *y*_f_. The right part of the plot indicates the correlation of the reference NF scores *y*_e_ and *y*_f_ vs. the corresponding reference bi-modal NF scores *y*_c_(= *y*_e_ + *y*_f_).

The results of the model validation of the NF-predictors (i.e., the learned activation patterns $\hat{\alpha }$ are applied to the learning set) are shown on the left side of Figure 4.

Pearson's correlation coefficients between the prediction and the ground truth are computed for each of the three learning sessions and for all subjects. Correlations are very high (> 0.8) for the two NF-predictors of *y*_c_ scores, indicating that the model is adapted to the problem of NF prediction. Indeed, the model could fit NF-fMRI scores using only EEG signal information with a median correlation of *r* = 0.81 with *y*_f_.

**Figure 5:**
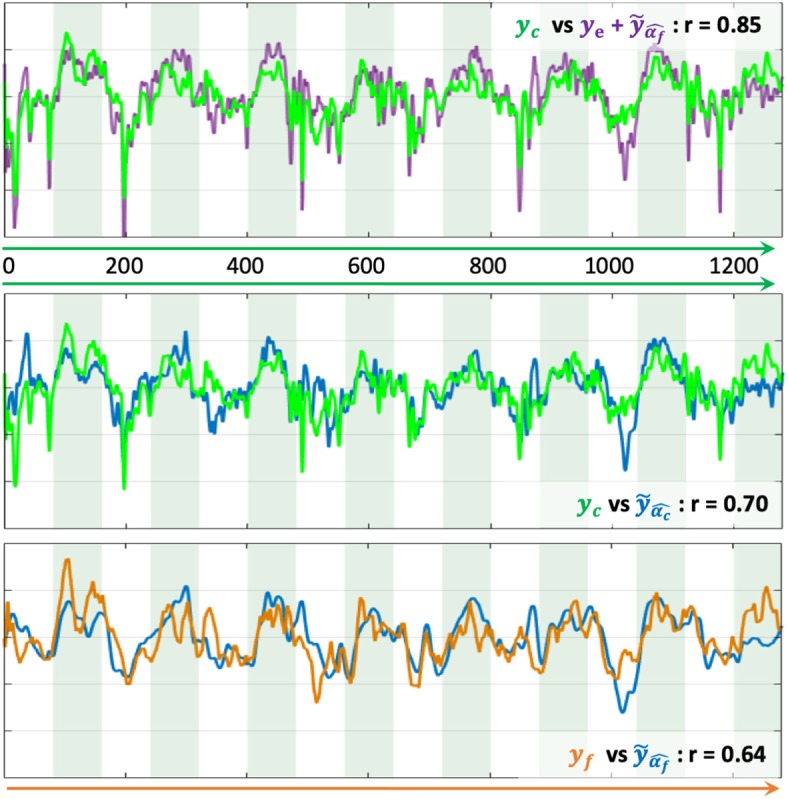
Examples of prediction of NF scores. The x-axis is the temporal axis in milliseconds. Vertical bands indicate the rest and task blocks. The correlation coefficient *r* indicates the correlation between each pair of time-series NF scores. (Top) Prediction of *y*_c_ with $y_{\text{e}}+{\tilde{y}}_{{\hat{\alpha }}_{f}}$. (Middle) Prediction of *y*_c_ with ${\tilde{y}}_{{\hat{\alpha }}_{C}}$. (Bottom) Prediction of *y*_f_ with ${\tilde{y}}_{{\hat{\alpha }}_{f}}$.

For the evaluation of the model prediction, the learned activation patterns are applied to the testing sets, i.e., the two other unseen NF sessions for each one of the three NF sessions. The significance of the differences between the correlations to the reference NF scores is assessed by applying a Fisher transformation to the correlation coefficients. The *p*-values obtained via Student's *t*-test after this transformation indicate the significance of the differences between the correlation coefficient distributions. Table 1 shows that $y_{\text{e}}+{\tilde{y}}_{{\hat{\alpha }}_{f}}$, whose median correlation is *r* = 0.74, better predicts *y*_c_ than *y*_e_ only, despite the fact that *y*_e_ is part of *y*_c_. The paired *t*-test gives a *p*-value *p* = 6.6e-4 (*t* = 3.52), meaning that the prediction of NF-fMRI scores by ${\tilde{y}}_{{\hat{\alpha }}_{f}}$ significantly adds information to *y*_e_. Predicting *y*_c_ scores with $y_{\text{e}}+{\tilde{y}}_{{\hat{\alpha }}_{f}}$ is also better than ${\tilde{y}}_{{\hat{\alpha }}_{C}}$ (*p* = 2e-42, *t* = 23.17), which is expected since the models predicts NF-fMRI scores and directly uses NF-EEG scores (with all the potential noise coming from the EEG measures), which are part of the reference score, as *y*_e_ is in $y_{\text{e}}+{\tilde{y}}_{{\hat{\alpha }}_{f}}$, and therefore correlates well with *y*_c_. Furthermore, the proposed model is able to predict *y*_f_ scores with a fair correlation of 0.36 in the median and 0.35 in the average. Thus, predicting NF-fMRI scores instead of predicting bi-modal NF scores seems to be the best way of predicting those bi-modal NF scores. Figure 5 gives examples of predictions of *y*_c_ with $y_{\text{e}}+{\tilde{y}}_{{\hat{\alpha }}_{f}}$ and ${\tilde{y}}_{{\hat{\alpha }}_{C}}$. It also illustrates that, even if the correlations of ${\tilde{y}}_{{\hat{\alpha }}_{C}}$ are lower than $y_{\text{e}}+{\tilde{y}}_{{\hat{\alpha }}_{f}}$ (Table 2), ${\tilde{y}}_{{\hat{\alpha }}_{C}}$ can correctly predict the reference score *y*_c_.

**Table 1:** Model prediction: Pearson's correlation coefficients, over all subjects and sessions, between NF-predictors and *y*_c_.

	**Paired** ***t*****-test**		
**Correlation (yc,.)**	**${\tilde{y}}_{{\hat{\alpha }}_{c}}$**	**$y_{\text{e}}+{\tilde{y}}_{{\hat{\alpha }}_{f}}$**	**y**_**e**_
${\tilde{y}}_{{\hat{\alpha }}_{c}}$	N.A.	*t* = −23.17	*t* = −12.76
		*p* = 1	*p* = 1
$y_{e}+{\tilde{y}}_{{\hat{\alpha }}_{f}}$	*t* = 23.17	N.A.	*t* = 3.52
	*p* = 2.e-42		*p* = 6.6e-4
*y*_e_	*t* = 12.76	*t* = −3.51	
	*p* = 9e-23	*p* = 1	N.A.

*The table shows p-values of a t-test in which the hypothesis is: models in columns correlate better with *y*_c_ than models in rows*.

**Table 2:** Sparsity of the learned models ${\hat{\alpha }}_{c}$ and ${\hat{\alpha }}_{f}$.

	**${\hat{\alpha }}_{0}$**	**${\hat{\alpha }}_{3}$**	**${\hat{\alpha }}_{4}$**	**${\hat{\alpha }}_{5}$**	**All**
${\hat{\alpha }}_{f}$	x	0.92 ± 0.03	0.96 ± 0.03	0.91 ± 0.03	0.93 ± 0.04
${\hat{\alpha }}_{c}$	0.87 ± 0.05	0.92 ± 0.03	0.96 ± 0.02	0.93 ± 0.03	0.92 ± 0.05

*Proportion of zero coefficients in the learned models*.

During the learning session (i.e., the NF session used to learn the predictor), subjects might unevenly focus on their fMRI or EEG feedbacks, and when subjects focus more on NF-fMRI than on NF-EEG, the EEG-signals might lose coherence with respect to the NF-fMRI scores. Despite this, the EEG signals could predict NF-fMRI scores with a correlation of 0.36 in median and 0.35 in mean (cf Figure 4, which is a fair correlation between such different modalities. Examples of NF prediction are given at the bottom of Figure 5, the plot shows the prediction ${\tilde{y}}_{{\hat{\alpha }}_{f}}$ of NF-fMRI scores on a testing set.

#### 4.2.2. Activation Patterns

We now focus on the learned models to evaluate the sparsity over sessions and subjects and observe the dispersion of the learned patterns over the sessions and frequency bands of a subject. For each subject and each learning session, we computed the proportion of zero coefficients of the activation patterns (Table 2). By construction of the design matrix ***X***_*c*_, ${\hat{\alpha }}_{c}$, and ${\hat{\alpha }}_{f}$ can be split into different activation patterns, as shown in the columns of Table 2. The models could select relevant coefficients of the design matrices to predict reference NF scores. In average, there are 87 non-zero coefficients on ${\hat{\alpha }}_{c}$ to predict *y*_c_ and 57 non-zero coefficients on ${\hat{\alpha }}_{f}$ to predict *y*_f_.

**Figure 6:**
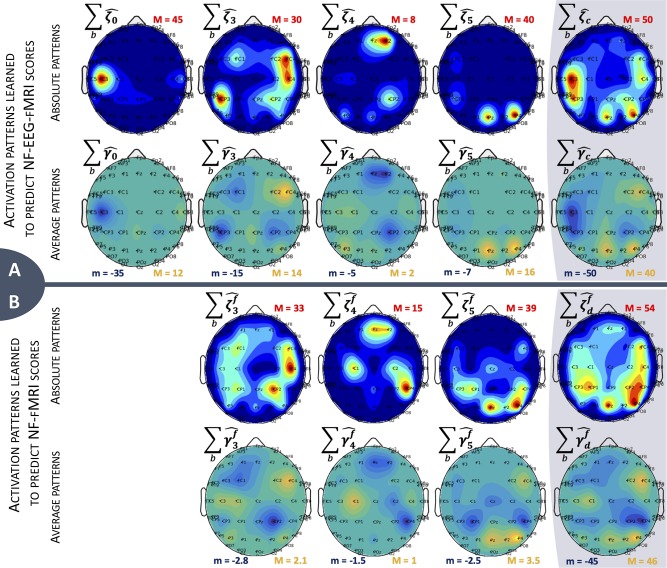
Activation patterns over sessions and frequency bands *b* for one example subject. There are two rows for each model; **(A)** represents activation patterns for ${\hat{\alpha }}_{c}$, and **(B)** represents activation patterns for ${\hat{\alpha }}_{f}$. The last column is the sum of the other ones. (Top row) Heat map representing the distribution of non-zero coefficients. Maximum value is indicated for each map with the red letter M, dark blue areas represent zero values. (Bottom row) Average activation patterns, representing the sign of the main non-zero values across subject and sessions. Minimum and maximum values are indicated for each map with the letters m and M, green areas represent zero values.

To display the activation patterns of a subject over their three sessions, we denote ${\hat{\zeta }}_{c}=\overset{3}{\underset{s=1}{\sum }}|{\hat{\alpha }}_{c}^{\left(s\right)}|\in {\mathbb{R}}^{M\times B}$ the absolute activation pattern of ${\hat{\text{α}}}_{c}$ (respectively, ${\hat{\zeta }}_{d}^{f}=\overset{3}{\underset{s=1}{\sum }}|{\hat{\alpha }}_{d}^{\left(s\right)}|\in {\mathbb{R}}^{M\times B}$ for ${\hat{\alpha }}_{f}$) and ${\hat{\gamma }}_{c}=\overset{3}{\underset{s=1}{\sum }}{\hat{\alpha }}_{c}^{\left(s\right)}\in {\mathbb{R}}^{M\times B}$ the average activation pattern (respectively ${\hat{\gamma }}_{d}^{f}=\overset{3}{\underset{s=1}{\sum }}{\hat{\alpha }}_{d}^{\left(s\right)}\in {\mathbb{R}}^{M\times B}$). We can display heat maps for each of the four absolute activation patterns at each electrode $\underset{b\in B}{\sum }{\hat{\zeta }}_{0},\underset{b}{\sum }{\hat{\zeta }}_{3}$, $\underset{b}{\sum }{\hat{\zeta }}_{4}$, and $\underset{b}{\sum }{\hat{\zeta }}_{5}$ (Figure 6, top line of each panel) and color maps of the four average patterns $\underset{b}{\sum }{\hat{\gamma }}_{0},\underset{b}{\sum }{\hat{\gamma }}_{3}$, $\underset{b}{\sum }{\hat{\gamma }}_{4}$, and $\underset{b}{\sum }{\hat{\gamma }}_{5}$ (Figure 6, bottom line of each panel) showing the sign of the strongest and most stable coefficients across all subjects and sessions. The activation patterns displayed at Figure 6 represent the dispersion of the learned parameters for one example subject over their three bi-modal NF sessions for which they received a bi-dimensional display. The subject used had a median correlation with the corresponding reference score of 0.44 for ${\tilde{y}}_{{\hat{\alpha }}_{f}}$, of 0.76 for ${\tilde{y}}_{{\hat{\alpha }}_{C}}$, and of 0.81 for $y_{\text{e}}+{\tilde{y}}_{{\hat{\alpha }}_{f}}$. All maps present different distributions of the non-zero coefficients. As expected when learning *y*_c_ scores, the most intense heat map $\underset{b}{\sum }{\hat{\zeta }}_{0}$ with a maximum value of 45 concentrates its non-zero values on the C3 channel (above the right-hand motor area) and corresponds to the design matrix ***X***_0_, directly extracted from the EEG signal without non-linear temporal delay. The next heat map in intensity order is $\underset{b}{\sum }{\hat{\zeta }}_{5}$ with two peaks with a parietal positioning above the Pz and P4 channels, corresponding to Brodmann area 7, which is involved in visuo-motor coordination. As presented in an interesting study (Sitaram et al., 2017), this brain area is active during generalized neurofeedback when feedback is presented visually. In Sitaram et al. (2017), it is also mentioned that this part of the cortex is part of the executive control network connected to the thalamus. The activation of the executive control network during motor imagery tasks is relevant and indicates that the subject is trying to do the task. It is also interesting to observe that the main activation peaks of $\underset{b}{\sum }{\hat{\zeta }}_{5}$ (Pz and P4) have opposite signs to the activation peak of $\underset{b}{\sum }{\hat{\zeta }}_{0}$ (above C3), suggesting a negative correlation between neural activation measured at C3 and the neural activation of the posterior parietal Pz and P4 channels, part of the control network in neurofeedback. Figure 6B presents comparable learned activation patterns to predict ${\tilde{y}}_{{\hat{\alpha }}_{f}}$, but more spread over sessions than for the model on panel A, even if the model has around 93% of null coefficients (Table 2). This suggests that, even for a single subject, the relation between EEG and fMRI changes over sessions, probably depending on the strategy used by the subject.

**Figure 7:**
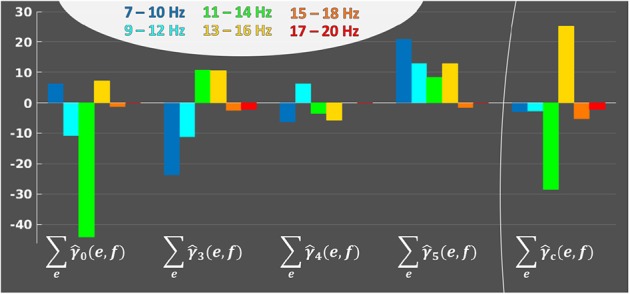
Frequency profiles, across subjects and sessions, of each average activation pattern, to represent the implication of each frequency band in the activation patterns. *e* ∈ {1, …, *E*}. For each average activation pattern, the y-axis indicates the sum of weights over electrodes for each frequency band *f* ∈ {1, …, *B*} on the horizontal axis.

At last, it is also possible to display the frequency profile of each average activation patterns. The 4 frequency profiles are $\underset{e\in \left\{1,\ldots ,E\right\}}{\sum }{\hat{\gamma }}_{0},\underset{e}{\sum }{\hat{\gamma }}_{3}$, $\underset{e}{\sum }{\hat{\gamma }}_{4}$ and $\underset{e}{\sum }{\hat{\gamma }}_{5}$, *e* ∈ {1, …, *E*}, summing weights over electrodes. Figure 7 shows that the most used frequency bands over all sessions and electrodes are the alpha band ([8–12] Hz) and lower beta band ([13–17] Hz); the last four frequency bands are not displayed as they only have null coefficients. We can observe the effect of ℓ_2_ regularization, which allows continuity in the frequency bands, and the effect of the subsequent ℓ_1_ regularization, which removed the smaller coefficients located in the high frequencies. When considering all activation patterns (Figure 7 right side), there is a change of sign between alpha and lower beta. Each activation pattern shows a different frequency profile, which, together with Figure 6, tends to demonstrate that patterns have complementary information.

### 4.3. Ablation Study

We run an ablation study to understand the impact of the non-linearity part of the model, obtained by the convolution of different HRFs inducing different delays for the prediction of NF-fMRI scores, by analyzing the results of the model without HRF convolutions. Therefore, in this ablation study, we used the exact same processes as described in section 4 (with a learning step and a testing step), except that we used the design matrix ***X***_0_ alone to learn the model parameters to predict *y*_c_ scores and *y*_f_ scores.

**Figure 8:**
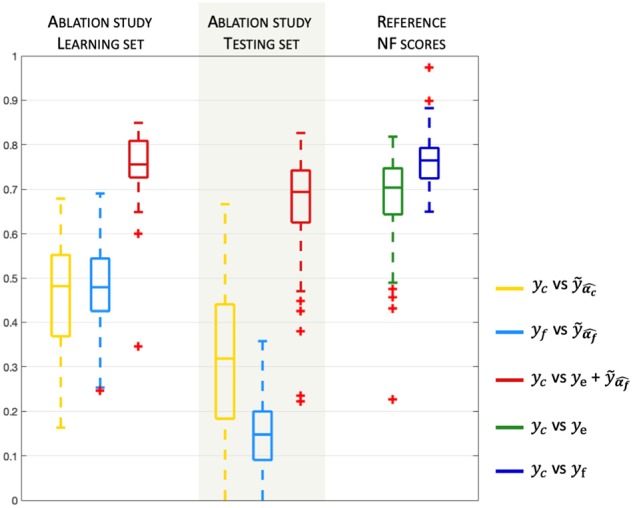
Ablation study. Removing non-linearity, keeping *X*_0_ only in the model to understand the importance of the non-linearity induced by the use of different HRF functions.

The results of the ablation study are displayed at Figure 8 and are to be compared with the results of the proposed model presented at Figure 4. Figure 8 shows that during the learning step, the prediction of ${\tilde{y}}_{{\hat{\alpha }}_{f}}$ using *X*_0_ only correlates with *y*_f_ with a correlation of only 0.48 in mean and median. This prediction is significantly improved by the use of the different HRF functions (paired *t*-test, *t* = −14, 64, *p* = 1*e*-26), as, in the proposed model, ${\tilde{y}}_{{\hat{\alpha }}_{f}}$ correlates with *y*_f_ with a correlation of 0.81 in median and mean. Therefore, it is natural to observe that the prediction of the bi-modal NF score by $y_{\text{e}}+{\tilde{y}}_{{\hat{\alpha }}_{f}}$ is also significantly improved by the use of non-linearity (paired *t*-test, *t* = −11, 87, *p* = 4*e*-21). Figure 8 shows that, for the testing step, the prediction of ${\tilde{y}}_{{\hat{\alpha }}_{f}}$ using *X*_0_ only correlates with *y*_f_ with a correlation of 0.14 in mean and 0.15 in median, which is significantly lower than with the proposed model (paired *t*-test, *t* = −9.56, *p* = 2*e*-18). The prediction of the bi-modal NF scores by $y_{\text{e}}+{\tilde{y}}_{{\hat{\alpha }}_{f}}$ is also significantly lower than with the proposed model (paired *t*-test, *t* = −2.74, *p* = 3*e*-3). Also, when using *X*_0_ only, the prediction of the bi-modal NF scores using $y_{\text{e}}+{\tilde{y}}_{{\hat{\alpha }}_{f}}$ does not significantly improve over *y*_e_ alone (paired *t*-test, *t* = −0.69, *p* = 0.5).

## 5. Discussion and Conclusion

The model validation supports that the optimization strategy we chose for our problem is adapted to the model, as is the choice of the different design matrices. The ablation study supports the use of different non-linear delays to improve the prediction of NF-fMRI scores using the EEG signal. The evaluation of the model prediction strongly suggests that predicting only NF-fMRI scores from an EEG signal while applying a Laplacian on the EEG signal appears to be the best solution. Indeed, for ${\tilde{y}}_{{\hat{\alpha }}_{C}}$, when predicting the bi-modal NF scores, the variability between EEG signals induces a decreasing correlation with the reference NF score *y*_c_. Also, NF-EEG scores can always be computed from available EEG signals, which leaves only NF-fMRI scores to be estimated from EEG signals. However, one might want to improve the selection of features for the computation of NF-EEG scores, but this raises different questions about the validation and the reference score. Here, we assumed that the given NF scores are relevant to the task and good enough to be considered as reference scores. As expected, for the prediction of the bi-modal NF scores by ${\tilde{y}}_{{\hat{\alpha }}_{C}}$, when decomposing the activation pattern ${\hat{\alpha }}_{c}$ (from ${\tilde{y}}_{{\hat{\alpha }}_{C}}$) into the four matrices corresponding to the different design matrices, the weights corresponding to ***X***_0_ (0 s of delay) are mainly located above C3, which is the center of the Laplacian used for the computation of the NF-EEG scores. This seems to support the fact that with a delay of 0 s, only the part of the NF coming from the EEG provides information.

A possibility to improve the prediction of NF-fMRI scores would be to use more NF sessions as learning sessions, since, as observed, EEG signals bring variability into the prediction. Each new bi-modal neurofeedback session could be added to the subject-specific model to better adapt the model to the subject or patient. This will be investigated in the next study. Given the improved correlation of the proposed NF predictor with bimodal NF scores, it would be interesting in future work to validate its improved performance in actual NF sessions compared to classical NF-EEG scores. In particular, to assess the response of subjects to the predicted bi-modal NF scores and particularly the predicted NF-fMRI scores learned by the proposed model over a standard NF-EEG neurofeedback session, a new and large enough study is needed, as subjects can learn at different paces to regulate their own brain activity.

Presently, the proposed model learns an individual or specific model for each subject, which allows a personalized model for adapted neurofeedback sessions. Also, a change in strategy for the task (here, no specific strategy for imagining moving their right hand was given to the subjects) might impact the learned model, as the relation between the EEG and fMRI signals may change. However, in a forthcoming work, we are investigating an adaptation of the methodology for the extraction of a common model, taking into account the differences between sessions and subjects, allowing the prediction of NF-EEG-fMRI scores on new subjects who did not participate in the model construction. The model might be less specific, but this would give access to neurofeedback sessions of bi-modal quality using EEG only for subjects with MRI contraindications and/or drive a subject-specific model estimation, respecting the strategy used by the subject to progress in their neurofeedback task. The learned NF predictor is, of course, specific to the particular task considered during the learning session. One can expect to generalize the approach to other tasks where spatial sparsity is relevant. Its extension beyond such tasks is more challenging.

Other ways to improve the method proposed here would be to investigate the use of dynamic functional connectivity, a relatively recent field in BOLD fMRI, the use of which along with EEG data requires further investigation (Tagliazucchi and Laufs, 2015). Dynamic functional connectivity would allow the different networks (reward, control, and learning) involved during neurofeedback sessions to be taken into account (Sitaram et al., 2017). Dynamic functional connectivity studies the temporal fluctuations of the BOLD signal across the brain and appears to be a promising approach in the EEG-fMRI research field (Allen et al., 2018). However, one should be careful with the potential unknown remaining noise coming from the MRI during simultaneous EEG-fMRI recording, which might unsettle the EEG signal coherence between electrodes.

The long-term objective of our project is to learn from EEG-fMRI NF sessions to provide, outside the MRI scanner, enhanced NF-EEG sessions (Figure 1). Future work will investigate the portability of the learned model (on EEG-fMRI neurofeedback data), outside the MRI scanner, introducing new challenges, such as dealing with the remaining noise in the MRI after artifact correction and the absence of ground truth once the EEG is measured outside the MRI scanner.

To conclude, the model proposed here is able to provide a good enough prediction of the NF-fMRI scores to overcome the absence of NF-fMRI and allows the estimation of NF-EEG-fMRI scores when using EEG only to be significantly improved.

## Data Availability Statement

Publicly available datasets were analyzed in this study. This data can be found here: https://openneuro.org/datasets/ds002338.

## Ethics Statement

The studies involving human participants were reviewed and approved by Comité de Protection des Personnes Ouest V Rennes. The patients/participants provided their written informed consent to participate in this study.

## Author Contributions

CC was the guarantor of integrity of the entire study. All authors contributed to the study concepts and design, data analysis and interpretation, and manuscript drafting or manuscript revision for important intellectual content. All authors gave approval of the final version of the submitted manuscript.

### Conflict of Interest

The authors declare that the research was conducted in the absence of any commercial or financial relationships that could be construed as a potential conflict of interest.
